# Inhibition of Interleukin-6-Induced Matrix Metalloproteinase-2 Expression and Invasive Ability of Lemon Peel Polyphenol Extract in Human Primary Colon Cancer Cells

**DOI:** 10.3390/molecules26237076

**Published:** 2021-11-23

**Authors:** Valentina Pagliara, Marina De Rosa, Paola Di Donato, Rosarita Nasso, Antonio D’Errico, Francesca Cammarota, Annarita Poli, Mariorosario Masullo, Rosaria Arcone

**Affiliations:** 1 Dipartimento di Scienze Motorie e del Benessere, Università degli Studi di Napoli “Parthenope”, 80133 Napoli, Italy; valentina.pagliara83@gmail.com (V.P.); rosaritanasso@gmail.com (R.N.); anto.derrico1994@gmail.com (A.D.); 2 Dipartimento di Medicina Molecolare e Biotecnologie Mediche, Università degli Studi di Napoli Federico II, 80131 Napoli, Italy; marina.derosa@unina.it (M.D.R.); francesca.cammarota88@gmail.com (F.C.); 3 CEINGE-Biotecnologie Avanzate, 80145 Napoli, Italy; 4 Dipartimento di Scienze e Tecnologie, Università degli Studi di Napoli “Parthenope”, 80143 Napoli, Italy; pdidonato@uniparthenope.it; 5 Istituto di Chimica Biomolecolare, Consiglio Nazionale delle Ricerche, 80078 Pozzuoli, Italy; apoli@icb.cnr.it

**Keywords:** matrix metalloproteinases (MMP)-2, interleukin-6, lemon (*Citrus limon*) peel extract, cell invasiveness, human primary colon cancer cells

## Abstract

Among matrix metalloproteinases (MMPs), MMP-9/2 are key enzymes involved in the proteolysis of extracellular matrices in the inflammatory process and in cancer. Since MMP-9/2 expression levels, activity, and secretion is up-regulated during inflammation in response to pro-inflammatory cytokines, such as interleukin-6 (IL-6), many efforts have been devoted to identifying factors that could inhibit the IL-6-induced MMP-9/2 expression. Up to now, several reports indicated that polyphenols from fruits and vegetables are among the major components of health promotion for their antioxidant properties and also for their anti-inflammatory and anti-cancer agents. Among plant derived polyphenols, lemon (*Citrus limon*) peel extract (LPE) shows anti-cancer properties in various cancer types. In our previous work, we demonstrated that LPE can reduce IL-6-induced migration/invasiveness and MMP-9/2 up-regulation in some gastric cancer cell lines. This study aims to exploit the anti-cancer properties of LPE using an in vitro system model of inflammation, consisting of IL-6-exposed human primary colon cancer cells. We first analyzed the effect of LPE on IL-6-induced cell migration and invasiveness by wound healing and Boyden chamber assay, respectively. The MMP-2 mRNA expression levels and gelatinolytic activity in the cell culture media were determined by q-PCR analysis and gelatin zymography, respectively, and finally, the effects of LPE on IL-6-induced JAK2/STAT3 signaling pathways have been investigated by Western blotting analysis. Our results show that LPE is able to inhibit the IL-6-dependent cell migration and invasiveness associated with the up-regulation of MMP-2 expression levels and that these effects are correlated to the STAT3 phosphorylation in human primary T88 and T93 colon cancer cells.

## 1. Introduction

Colorectal cancer (CRC) is one of the most common cancers with a short overall survival and cancer-related mortality rate that shows a variability depending on the age, the gender, and the country of the patient [1]. The differential incidence among the different geographic areas in the world has not only been attributed to genetic risk factors but also to socioeconomic status, including dietary factors, and body composition [2]. Colorectal tumors are characterized by a great heterogeneity of the phenotype and genotype and are greatly influenced by the immune system and the cellular microenvironment [3]. The degradation of the extracellular matrix (ECM) represents a crucial step in the invasion and migration of cancer cells, and this process is strictly dependent on the activity of numerous enzymes. Matrix metalloproteinases (MMPs) are the major enzymes responsible for the degradation of collagen and proteins in the ECM [4,5]. Among the different MMPs, MMP-9 and MMP-2 belong to a family of zinc-dependent endo-peptidases which are secreted by stromal and tumor cells as inactive zymogens, and then activated following the cleavage of the pro-domain peptide into their active form [5]. MMP-2 and MMP-9, known as gelatinase A and B, are critically involved in tumor invasion and metastasis and their expression has been associated with poor overall survival in patients with colon cancer [6,7]. A close correlation has been demonstrated between the inflammatory state and increased expression of MMPs, which is caused by proinflammatory cytokines, including interleukin-6 (IL-6) [8,9]. Interleukin-6 (IL-6) is a major inflammatory cytokine also involved in cancer and autoimmune diseases [10]. Numerous evidences have shown that IL-6 induces the migration and invasiveness of different types of tumor cells [11,12,13,14] and therefore represents a prognostic factor related to cell survival [15]. In fact, elevated levels of IL-6 have been detected in the plasma of gastric and colon cancer patients and in gastric and colon cancer cell lines, confirming the role of IL-6 as a predictor of poor prognoses and as correlated to tumor aggressiveness [8,16,17]. The molecular mechanism activated by IL-6 involves the activation of JAK/STAT3 signaling [18], which leads to the transcriptional activation of numerous target genes through the action of STAT3, which then results in tumor proliferation and/or survival. Furthermore, STAT3 also regulates the expression of factors that promote angiogenesis and cell invasiveness, including MMPs [19,20,21]. In this context, IL-6 behaves as a pro-metastatic agent as described in the human gastric cancer AGS and MKN-28 cell lines [22,23,24]. Among the various factors involved in the onset of CRC, lifestyle and diet are also considered [25]. Polyphenols in fruit and vegetables are among the main components of health for their antioxidant properties and also for their anti-inflammatory and antitumor agents [26,27,28]. In our previous studies, we have shown that the polyphenols of plant origin contained in the extract from the flesh of Annurca apples and lemon (*Citrus limon*) peel extracts (LPE) are able to reduce the migration/invasiveness induced by IL-6 or on the up-regulation of MMP-9/2 in MKN-28 and AGS cancer cells [24,29].

In light of this previous evidence, in this study, we have analyzed the effects of LPE using primary human colon T88 and T93 cancer cells, which have been previously characterized [30,31,32]. These primary colon cancer cells were isolated and established from the tumor tissues of two patients affected by sporadic colon adenocarcinoma. The T93 cell line exhibited a chromosomal instability (CIN) phenotype and the T88 cell line exhibited a microsatellite instability (MSI) phenotype. It has been previously demonstrated that these cells both underwent EMT from epithelial adenocarcinoma cells; indeed, they simultaneously expressed epithelial and mesenchymal markers, such as cytokeratin and E-cadherin, and Vimentin and N-cadherin, respectively, together with high levels expression of EMT-associated transcription factors, stemness markers, and the Cyclooxygenase-2 (Cox-2) enzyme [30,31,32].

The protective effect of LPE against the increase in cell migration and invasiveness induced by IL-6 was evaluated by pretreatments with LPE before exposure to IL-6. The effects of LPE on IL-6-induced JAK2/STAT3 signaling pathways were also analyzed. Our results indicate that LPE is capable of inhibiting rIL-6-dependent migration and invasiveness associated with up-regulation of MMP-2 expression levels in human colon carcinoma cell lines T88 and T93.

## 2. Results

### 2.1. The LPE Pretreatment Reduces the rIL-6-Induced Migration and Invasiveness of Human Primary T88 and T93 Colon Cancer Cells

**Figure 1:**
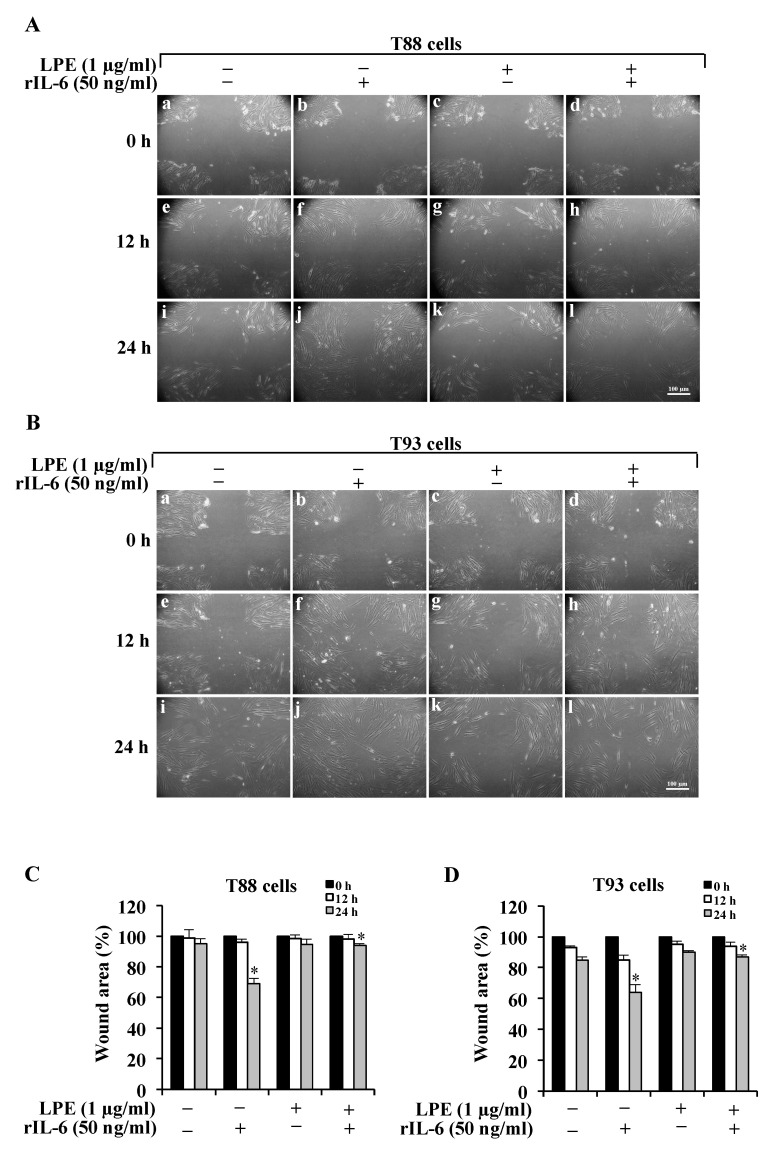
Effect of rIL-6 exposure with or without LPE pretreatment on the cell migration of T88 and T93 cells by wound healing assay. Representative images of (**A**) T88 and (**B**) T93 cells captured at time 0, 12, and 24 h by a phase-contrast microscope (10× objective). Cells kept in serum-free DMEM (**a**,**e**,**i**) were treated with rIL-6 alone (**b**,**f**,**j**), LPE alone (**c**,**g**,**k**), or pre-treated with LPE (1 μg/mL GAE) for 6 h and then the medium was removed and replaced with the same amount of fresh medium containing rIL-6 (50 ng/mL) for 12 h or 24 h. Quantitative analysis of the wound area of T88 (**C**) and T93 cells (**D**) at time 0, 12, and 24 h. For each treatment, the data shows the wound area at the indicated time in comparison to that of the open wound at time 0, set at 100%. Results are presented as mean ± SD (*n* = 3) (* *p* < 0.05, statistically significant vs. untreated cells).

Since several reports demonstrated that IL-6 enhances the migration ability and invasiveness of gastric and colon cancer cells [8,12,20,23,24,33], we investigated the effect of the treatment with rIL-6 on either migration or invasive ability in human primary T88 and T93 colon cancer cells. In addition, to explore whether LPE could counteract the rIL-6-dependent effects, T88 and T93 cells were first pretreated with LPE for 6 h and then stimulated with rIL-6 for a further 24 h. To avoid serum interference with LPE components, all the treatments were performed in serum-free media and, at the end of the incubation, the treated cells were compared with the untreated cells that were kept in serum-free conditions. The cell migration was evaluated by the wound-healing assay that is shown in Figure 1. As displayed in this figure, in both cells, the exposure to rIL-6 alone led to a significant reduction in the wound area (Panel A, B, images b, f, j) compared to that of untreated cells; this result clearly demonstrates that rIL-6 enhances the cell migration ability. In contrast, neither serum deprivation (Panel A, B, images a, e, i) nor treatment with LPE alone (Panel A, B, images c, g, k) affected the wound area, compared to that of untreated cells (Panel A, B, images a, e, i) in both T88 and T93 cells. Quantitative analysis of the wound area (Panel C, D) shows that in both cells, the exposure to rIL-6 induces minimal reduction in the wound area at 12 h, whereas ~40% reduction is observed at 24 h, compared to that of cells kept in serum-free conditions or exposed to LPE alone. It is noteworthy that the pre-treatment with LPE is able to inhibit the rIL-6-induced cell migration, as demonstrated by the wound area that is similar to that of control cells, either in T88 (Panel A, images d, h, l) or in T98 (Panel B, images d, h, l). A maximal effect (~30%) was observed at 24 h and was compared with the wound area of cells stimulated with rIL-6 alone.

**Figure 2:**
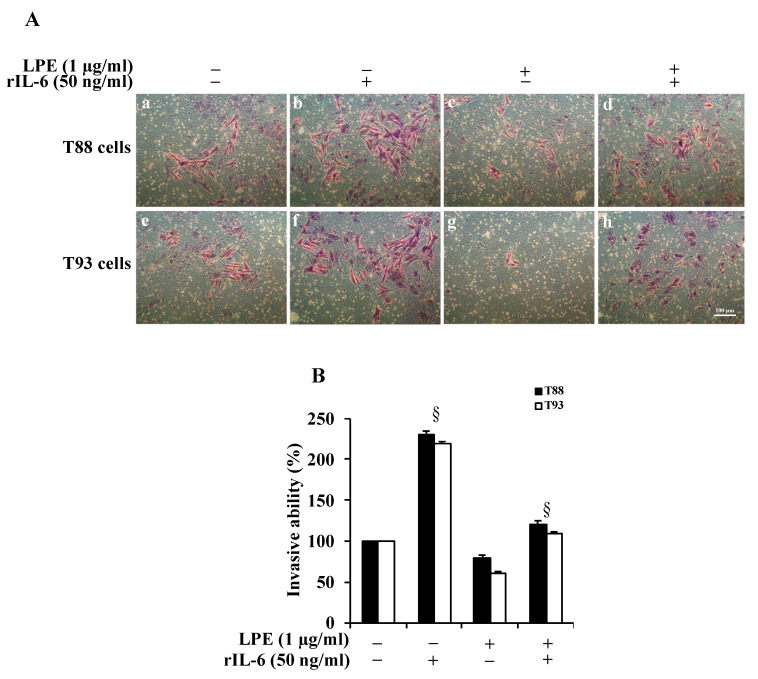
Effect of rIL-6 exposure with or without LPE pretreatment on the invasive ability of T88 and T93 cells. The cells were seeded in trans-well chambers for the Matrigel invasion assay, and then treated with rIL-6 alone (**b**,**f**), LPE alone (**c**,**g**), or pre-treated with LPE (1 μg/mL GAE) for 6 h. Then, the medium was removed and replaced with the same amount of fresh medium containing rIL-6 (50 ng/mL) for 24 h (**d**,**h**). Control cells were kept in serum-free DMEM (**a**,**e**). Thereafter, the cells at the bottom of the filters were fixed, stained, and observed by a phase-contrast microscope (10× objective). (**A**) Representative photomicrographs of random fields of T88 and T93 cells subjected to the different treatments. (**B**) The cell invasiveness was determined by the cell count in five fields, randomly selected, per membrane. Quantification was relative to untreated cells, cultured in serum-free DMEM, set at 100%. Results are presented as mean ± SD (*n* = 3) (§ *p* < 0.01, statistically significant vs. untreated cells).

To evaluate the effect of rIL-6 alone or after LPE pretreatments on T88 and T93 cell invasiveness, we performed a Matrigel assay. Figure 2, Panel A reveals that, in both cells, rIL-6 induces an ~2-fold (Panel B) increase in the number of cells that had invaded through the membrane (Panel A, images b, f), compared to those found in untreated cells (Panel A, images a, e). The treatment with LPE alone for 24 h (Panel A, images c, g) slightly decreases the cell invasiveness (Panel B), compared to untreated cells. The LPE pre-incubation before rIL-6 exposure is able to reduce the cytokine-induced increase of cell invasiveness in both T88 and T93 cells (Panel A, images c, g); in fact, an ~2.5-fold decrease of invasive ability is observed in cells pretreated with LPE compared to that of cells exposed to rIL-6 alone (Panel B).

### 2.2. The LPE Pretreatment Reduces the rIL-6-Induced Up-Regulation of MMP-2 Activity and mRNA Expression Levels in T88 and T93 Cells

**Figure 3:**
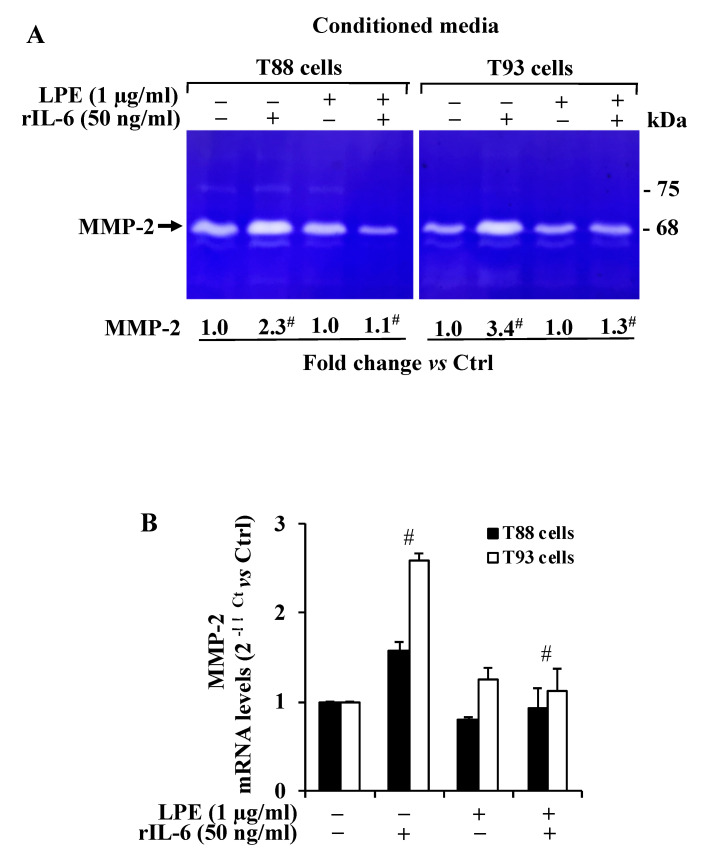
Effect of rIL-6 exposure with or without LPE pretreatment on MMP-2 gelatinase activity and mRNA expression levels. (**A**) Gelatinolytic activity in T88 and T93 cells revealed by gel zymography. Cells, pretreated with LPE (1 μg/mL GAE) for 6 h, were then exposed to rIL-6 (50 ng/mL) for a further 24 h. After the treatments, the conditioned media were collected, and the cells harvested for cell protein and mRNA preparation. Cell-conditioned media were concentrated by ultrafiltration, and volumes corresponding to 40 μg of cell proteins were analyzed under non-reducing conditions through a 12% SDS-polyacrylamide gel co-polymerized in the presence of gelatin (1 mg/mL). (**B**) MMP-2 mRNA expression levels in T88 and T93 analyzed by qPCR analysis. Expression levels of MMP-2 mRNA of the treated cells were calculated vs. the untreated cells, set to 1. Results are presented as mean ± SD (*n* = 3) (# *p* < 0.001, statistically significant vs. untreated cells).

As colorectal cancer progression involves MMP-2/9 activity that is up-regulated by IL-6, we first analyzed whether the treatment with rIL-6 alone or after the LPE pretreatment could affect the gelatinase activity in T88 and T93 cells. To this aim, gel zymography analysis was performed on cell-conditioned media from T88 and T93 cells subjected to the different treatments. The result (Figure 3A) revealed that the untreated T88 and T93 cells expressed a strong gelatinolytic activity corresponding to the MMP-2 molecular mass (~62–72 kDa active MPP-2 form), and therefore, these cells express high MMP-2 gelatinolytic activity levels; in contrast, no signal was detected at the molecular mass corresponding to MMP-9 (~82 kDa active MMP-9 form).

As shown by zymography, the exposure to rIL-6 alone resulted in an ~2.3 and 3.4-fold increase of MMP-2 signals, respectively, in T88 and T93, when compared to that of untreated cells.

In contrast, the conditioned medium from cells pre-treated with LPE (6 h) and then exposed to rIL-6 (24 h) shows a MMP-2 gelatinolytic activity that is similar to that of untreated cells, thus suggesting that the LPE pretreatment is able to prevent the rIL-6-dependent increase of MMP-2 activity. The zymography also shows that the exposure to LPE alone (24 h) does not alter the basal MMP-2 expression in treated cells when compared to untreated cells.

To assess whether the MMP-2 enzyme regulation by rIL-6 and LPE could also involve a MMP-2 gene transcriptional control, we also performed a quantitative analysis of MMP-2 mRNA level by qPCR of untreated and treated T88 and T93 cells. The result (Figure 3B) shows that rIL-6 induced an up-regulation of MMP-2 mRNA levels in both the cells, with a major induction effect in T93 cells. The LPE pretreatment appears to counteract the rIL-6-dependent MMP-2 mRNA up-regulation, with a similar reduction in both cells, that resembles the MMP-2 mRNA level observed in control cells. These results clearly demonstrate that rIL-6 up-regulates both MMP-2 enzyme activity and mRNA levels and that the LPE pretreatment counteracts the rIL-6-induced enhancement expression of MMP-2 expression in T88 and T93 cells.

### 2.3. The LPE Pretreatment Inhibits the rIL-6-Dependent STAT3 Phosphorylation Levels in T88 and T93 Cells

**Figure 4:**
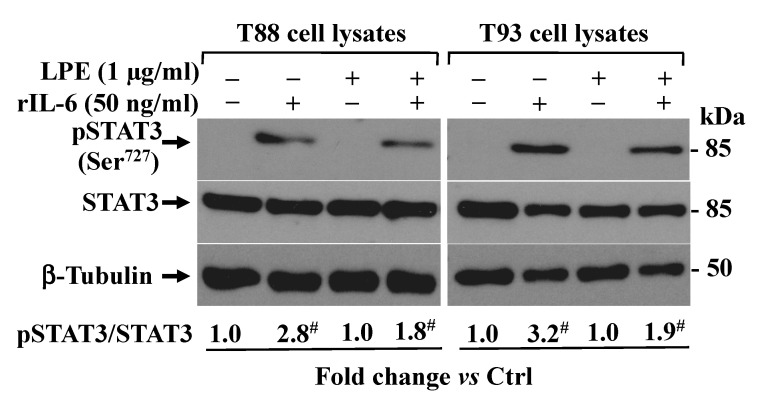
Effect of rIL-6 exposure with or without LPE pretreatment on STAT3 phosphorylation levels in T88 and T93 cells. Western blotting analysis of phospho-STAT3 protein expression levels in cells treated with rIL-6 alone (50 ng/mL for 24 h) or after preincubation with LPE (1 μg/mL GAE for 6 h)) and then exposed to rIL-6 (50 ng/mL for 24 h). The relative phospho-STAT3/STAT3 protein fold change level in the treated cells was calculated vs. the untreated cells, set to 1, is shown under each lane. Results are presented as mean ± SD (*n* = 3) (# *p* < 0.001, statistically significant vs. untreated cells).

Since STAT3 phosphorylation is a crucial event in the signaling pathway, triggered by IL-6 in MMP-2 up-regulation in gastric cancer cells [20,33], we analyzed the STAT3 phosphorylation status following the rIL-6 exposure before and after preincubation with LPE in T88 and T93 cells. The results obtained by Western blotting analysis (Figure 4) demonstrated that the rIL-6 exposure was able to induce an ~3-fold increase of STAT3 phosphorylation in Serine 727 residue (Ser^727^STAT3) levels, compared to that of untreated cells kept in a serum-free medium, which showed only a barely detectable level. A negligible amount of pSTAT3 protein levels was detected in LPE stimulated cells, thus suggesting that LPE alone does not affect the STAT3 phosphorylation status.

This result clearly indicates that the LPE pretreatment is able to prevent the rIL-6-dependent STAT3 phosphorylation and, therefore, that the LPE effects are also mediated through the IL-6-induced STAT3 signaling pathway in T88 and T93 cells.

## 3. Discussion

In this study, we demonstrated that the inflammatory cytokine rIL-6 affects the migration and the invasiveness of two human primary adenocarcinoma cells, namely, T88 and T93 cells [30,31,32], and that this effect is well-correlated with the up-regulation of MMP-2 enzyme activity and mRNA expression levels. These findings agree with the key role of this metalloproteinase in the degradation of the ECM components, a process involved in tumor metastasis and cancer.

It should be noted that, in our studies, the zymography analysis shows a very high MMP-2 gelatinolytic level, but not that of MMP-9, which does not appear as a detectable signal even in smaller bands derived from degraded products. In addition, we observe a strong correlation between MMP-2 gelatinolytic activity and mRNA expression levels among all the treatments groups.

This evidence is consistent with previous observations on gastrointestinal malignancy demonstrating that the overexpression of MMP-2 is related to the conversion from premalignant conditions to a malignant phenotype, whereas MMP-9 is overexpressed in premalignant polyps [34,35], suggesting that the latter is expressed in the early stage during the adenocarcinoma malignant transformation.

In this scenario, the presence of the inflammatory IL-6 cytokine induces a strong up-regulation of MMP-2 for both the protein and mRNA expression levels.

Furthermore, the data reported here show that LPE is able to inhibit the rIL-6-dependent cell migration and invasiveness in human primary T88 and T93 colon cancer cells [30,31,32] via the up-regulation of MMP-2 expression, and that the observed effects correlate with the STAT3 phosphorylation levels. The protective effects showed by plant polyphenols against different types of cancer [36] include their anti-inflammatory and antioxidant properties [26,27,28,29]. Our results are in agreement with those reported previously on the anti-inflammatory effects of diet polyphenols that are exerted mainly through the down-regulation of circulating IL-6 [37,38,39].

In our previous studies, we analyzed the effect of rIL-6 and LPE using the human gastric MKN-28 cancer cell line, and we found a similar LPE protective effect against rIL-6-dependent increases in cell migration-invasiveness [24]. However, the MKN-28 cells showed a great increase following rIL-6 treatment of both MMP-2 and MMP-9; conversely, in T88 and T93 cells, we only detected MMP-2 up-regulation in our experimental conditions. Therefore, these findings suggest a different scenario in the molecular and cellular mechanisms underlying the tumor spreading between gastric and colon cancer cells, in which MMP-2 and MMP-9 could play a different role and, also, they could represent specific tumor-related biomarkers. Furthermore, these results, in addition to extending the beneficial effects already reported for LPE [24,28] to human primary colon carcinoma cells, reinforce the protective effect exerted by this vegetables industry by-product extract in the prevention and/or progression of gastric-intestinal cancers. LPE polyphenols have already been the object of our interest since they are a valuable example of those biologically active compounds that can be obtained from a residue of the food chain production.

## 4. Materials and Methods

### 4.1. Materials

Dulbecco’s modified Eagle medium (DMEM), fetal bovine serum (FBS), trypsin-EDTA, and phosphate-buffered saline (PBS) pH 7.4 were obtained from Lonza (Basel, Switzerland); rIL-6 was produced as reported [18,24,40]. The chemiluminescent HRP substrate kit and the centrifugal filter units (Amicon Ultra 10K) were obtained from Merk Life Science, (MI), Italy.

Lemon peel polyphenol extract (LPE) was prepared as previously described [24], the total phenolic content was determined according to the adapted Folin–Ciocalteau colorimetric method [41], and the results were expressed as milligrams gallic acid equivalent (GAE) per gram of dry sample. The raw extracts were concentrated at 40 °C under vacuum by a rotary evaporator, and dissolved in the suitable solvent for the biological assays.

### 4.2. Cell Cultures and Treatments

The human primary T88 and T93 colon cancer cells were isolated and propagated as reported [30,31]. The cells were cultured in Dulbecco’s modified Eagle’s medium (DMEM, (Merk Life Science, (MI), Italy) supplemented with 10% heat-inactivated fetal bovine serum (FBS), 1.5 mM L-glutamine, 100 units/mL penicillin, and 100 μg/mL streptomycin under a humidified atmosphere of 5% CO_2_ at 37 °C. The treatments of sub-confluent cells with 50 ng/mL of rIL-6 [18,24,40] or lemon peel extract (LPE) (1 μg/mL) [24,28] were performed for 24 h in serum-free DMEM. To assess the LPE protective effect, the cells were pretreated with LPE (1 μg/mL) for 6 h; thereafter, the medium was removed and replaced with fresh medium containing rIL-6 (50 ng/mL) and the exposure prolonged for a further 24 h. To avoid serum interference with LPE components, all the treatments were performed under serum-free conditions and in the presence of 0.5% (v/v) final concentration EtOH, which was used as a LPE vehicle.

### 4.3. Wound Assay

The cell migration was evaluated by a wound assay [42]. Briefly, the cells (2 × 10^5^ cells/well) were seeded into a 6-well plate and incubated with complete medium at 37 °C and 5% CO_2_. After 24 h of incubation, the cells were scraped horizontally and vertically with a sterilized P10 pipette tip (Gilson), subjected to different treatments in medium with 0.5% FBS, and two views on the cross of each well were photographed at 0, 12, and 24 h using a Zeiss Axiovert 40 CFL inverted microscope (Carl Zeiss, (MI), Italy), 10× objective. The microscope was equipped with a 12.1-megapixel CCD digital video camera (Canon, PowerShot G9, Italy) with a digital image software (Remote Capture Biomolecules 2019, 9, 833 5 of 17 DC, Canon, (MI), Italy). Quantitative analysis of the scratch assay was performed by measuring the gap area using the free image-processing software ImageJ, version 1.47 (https://imagej.nih.gov/ij/download.html, accessed on 18 November 2021).

### 4.4. Matrigel Invasion Assay

The cell invasiveness was determined by cell invasion assay using a Boyden chamber coated with Matrigel (BioCoat Matrigel invasion chambers, cat. n. 354480, BD Bioscience, Bedford MA; 8 μm, BD Bioscience, (MI), Italy). The cell culture inserts were rehydrated and prepared as previously described [7,26]. Briefly, 2 × 10^4^ cells in 0.5 mL of DMEM with 0.5% FBS were seeded in the upper chamber, and 750 μL medium with 5% FBS was placed in the lower chamber. After 24 h, cells in the upper chamber were removed with the cotton swab, and the cells at the bottom of the filters were fixed and stained with a Diff-Quick kit (cat. n. B4132-1A, Becton-Dickinson). After two washes with water, the inserts were allowed to air dry, and phase-contrast images were captured as described above using an LD A-Plan 20 ×/0.30 Ph 1 objective. Cell invasive ability was determined by cell counting in five fields randomly selected per membrane.

### 4.5. RNA Extraction, Reverse Transcription (RT), and Quantitative Real-Time Polymerase Chain Reaction (qPCR)

Total RNA was purified by the ultrapure TRizol reagent (Gibco BRL, Life Technology Italia, (MB), Italy) according to the manufacturer’s instructions. The concentration and purity of RNA was determined spectrophotometrically by reading the absorbance at 260 and 280 nm. Aliquots (1 μg) of total RNA were subjected to DNase I digestion (Thermo Fisher Scientific, (MB), Italy) and reverse-transcribed using a SensiFAST ^TM^cDNA synthesis kit (Cat. N. BIO-65054, Bioline, Italy) according to the manufacturer’s protocol. Real-time PCR was carried out using the PowerUP SYBR Green Master Mix (Thermo Fisher Scientific, (MB), Italy), the Quant Studio 7 Flex instrument (Thermo Fisher Scientific, Monza, Italy), and the fast gene-expression method with the following conditions: a first denaturation step at 95 °C for 20 s, followed by 40 cycles at 95 °C for 1 s and 60 °C for 30; then, a melting curve analysis was performed, raising the temperature from 60 °C to 95 °C with a 0.5 °C/s increase. Reactions were carried out in triplicate, and the 18S gene was used as an internal control to normalize the variability in expression levels. The 2^−^^ΔΔCT^ (cycle threshold) method was used to calculate the results and mRNA expression levels were determined as fold-induction relative to control cells, set to 1. The human MMP-2 and 18S primers [24] were used for qPCR reactions that were carried out in triplicate; mRNA expression levels were determined as fold-induction relative to control cells, set to 1.

### 4.6. Western Blotting Analysis

Cells were seeded in 6-well plates (2 × 10^5^ cells/well) and subjected to different treatments. Whole-cell protein extracts were prepared by lysing cells in 50 mM Tris-HCl pH 8.0, 150 mM NaCl, 0.5% sodium deoxycholate, 0.1% SDS, 1 mM EDTA, 1% Igepal, 1× protease inhibitor (cat. n. 11836153001, Roche Applied Science, Monza (MB), Italy), and a phosphatase-inhibitor cocktail (cat. n. 524627, Calbiochem, (MI), Italy). Protein concentration was determined by a colorimetric assay [43]. The culture medium was harvested and contaminating cells and debris were removed by centrifugation at 6000× *g* for 20 min at 4 °C. The cell supernatant was then concentrated by approximately 20-fold by centrifugation at 5000× *g* using Ultra-4, PLGC Ultracell-PL Membrane, 10 kDa cut-off (cat. n. UFC 801024, Merck Millipore, (MI), Italy), at 4 °C. Whole-cell protein extracts (40 μg) and the appropriate volumes of concentrated conditioned media (corresponding to 30 μg total cell proteins) were heated at 95 °C for 5 min in Laemmli denaturing buffer in the presence of 2 M urea, and then loaded onto 12% reducing SDS-PAGE [44]. After electrophoresis, proteins were transferred to a PVDF membrane (GE Healthcare Life Sciences, (MI), Italy) that was incubated with a Ponceau S solution for protein staining; the membrane was then exposed to the primary antibody as reported [45]. The following antibodies were used: rabbit polyclonal antibody raised against phospho-STAT3 (Ser 727) (1:1000, Cat. N. 9134, Cell Signaling Technology, Euroclone, (MI), Italy); STAT3 (1:1000, Cat. N. 9139, Cell Signaling Technology, Euroclone, (MI), Italy); beta-tubulin III antibody (Cat. N. T2200, Sigma Aldrich, (MI), Italy) that was used as protein loading control. After incubation with the appropriate peroxidase-linked secondary antibody, an Immobilon Western Chemiluminescent HRP Substrate (ECL) kit (Cat. No. WBKLS0500, Merck Millipore, (MI), Italy) was used for visualization. Densitometric analysis of the ECL signal was performed using the free image-processing software ImageJ, version 1.47 (https://imagej.nih.gov/ij/download.html, accessed on 18 November 2021).

### 4.7. Gelatin Zymography

Gelatinolytic activity in the cell-conditioned medium was determined by SDS-PAGE zymography, as described previously [7,26]. Samples were analyzed under non-reducing conditions without boiling, through a 12% SDS-polyacrylamide gel co-polymerized in the presence of gelatin (1 mg/mL, cat. n. G1890, Sigma-Aldrich, (MI), Italy). Electrophoresis was conducted at 35 mA for 90–120 min at 4 °C. After the run, the proteins in the gels were renatured in a 2.5% Triton X-100 solution for 1 h. The gels were then incubated with 50 mM Tris-HCl pH 7.5, 200 mM NaCl, 5 mM CaCl_2_, and 5 μM ZnCl_2_ at 37 °C for 48 h, which allows for substrate degradation. Finally, the gels were fixed in 30% methanol and 10% acetic acid for 30 min and stained with 0.5% Coomassie Brilliant Blue R-250. Proteolytic bands were visualized as clear bands against a blue background, after a de-staining step with 50% methanol and 5% acetic acid.

### 4.8. Statistical Analysis

The data were expressed as mean ± SD of at least three independent experiments performed in triplicate. The statistical significance of treated samples against control cells (cultured in serum-free medium) was determined by a one-way analysis of variance (ANOVA), followed by Bonferroni’s test (*p*-values: * *p* < 0.05; § *p* < 0.01; # *p* < 0.001).

## 5. Conclusions

In summary, our results demonstrated that MMP-2 plays a major role in cancer progression for its ability to degrade ECM components, and that its expression and activity are up-regulated through IL-6 in T88 and T93 cells. Furthermore, we demonstrated that LPEs behave as anti-inflammatory agents able to prevent or counteract the invasiveness of colon cancer cells, mainly by the inhibition of IL-6-dependent effects induced on MMP-2 activity and expression. Therefore, MMP-2, in addition to MMP-9, represents a potential target molecule for treating malignant colon tumors. The further exploitation of LPE biological activity also represents a possible valorization strategy of a residual biomass whose disposal represents an economical and environmental issue for the agro-industry sector. Therefore, the production of LPE could represent a fruitful strategy for the obtainment of bioactive agents for healthy promotion.

## Data Availability

The data presented in this study are available in the article.

## Sample Availability

Samples included in this article, can be requested to the corresponding authors.
